# Association between cognitive function and Cre/BW in middle-aged and older Chinese adults: evidence from the CHARLS

**DOI:** 10.3389/fpubh.2025.1494916

**Published:** 2025-03-26

**Authors:** Shuting Li, Mengya Qi, Yanxue Wang, Xingmeng Lu, Xingang Li

**Affiliations:** Beijing Ditan Hospital, Capital Medical University, Beijing, China

**Keywords:** CHARLS, mild cognitive impairment, Cre/BW, sarcopenia, Chinese

## Abstract

**Background:**

Cre/BW has been widely validated as a reliable biomarker for assessing muscle mass in clinical and epidemiological studies. Accumulating evidence from longitudinal cohort studies has demonstrated a significant association between sarcopenia and progressive cognitive decline in aging populations. To further elucidate this relationship, we conducted a comprehensive analysis using data from a nationally representative survey.

**Methods:**

This study utilized longitudinal data from the China Health and Retirement Longitudinal Study (CHARLS), with baseline measurements collected in 2012 and follow-up assessments conducted in 2018. To comprehensively evaluate the association between Cre/BW and cognitive function, we employed a dual analytical approach. Cross-sectional analyses were performed using multivariable-adjusted linear regression models for continuous cognitive scores and logistic regression models for dichotomous cognitive outcomes. For longitudinal assessment, we implemented time-to-event analyses using Cox proportional hazards models, with rigorous adjustment for potential confounders including demographic characteristics, lifestyle factors, and comorbidities.

**Results:**

Initial unadjusted linear regression analysis revealed a significant inverse association between Cre/BW ratio and total cognitive function score (*β* = −0.111, 95% CI: −0.013 to −0.008, *p* < 0.001). This association remained statistically significant after comprehensive adjustment for potential confounders, albeit with attenuated effect size (*β* = −0.052, 95% CI: −0.007 to −0.003, *p* < 0.001). When analyzing cognitive function scores by quartiles, we observed a consistent pattern where lower Cre/BW ratios were associated with better cognitive performance, even after multivariable adjustment (OR = 0.973, 95% CI: 0.951 to 0.996, *p* = 0.019). Longitudinal analysis using Cox proportional hazards models demonstrated that higher Cre/BW ratios were significantly associated with increased risk of cognitive impairment (HR = 1.207, 95% CI: 1.073 to 1.359, *p* = 0.002). Notably, participants in the highest Cre/BW quartile showed a 1.118-fold increased risk of cognitive impairment compared to those in the lowest quartile (95% CI: 1.048 to 1.346, *p* = 0.007), suggesting a dose–response relationship between Cre/BW ratio and cognitive outcomes.

**Conclusion:**

Our findings demonstrate a significant inverse association between Cre/BW and cognitive function in the general Chinese adult population. Longitudinal analysis revealed that elevated Cre/BW ratio serves as an independent risk factor for cognitive impairment, with this association persisting after extended follow-up and comprehensive adjustment for potential confounding factors.

## Introduction

Mild cognitive impairment (MCI) represents a transitional clinical state between normal age-related cognitive decline and dementia (1). Individuals with MCI exhibit measurable cognitive deficits, particularly in memory, executive function, language, praxis, and visuospatial skills, while maintaining relatively preserved activities of daily living compared to cognitively normal individuals of similar age and educational background (2). The neurocognitive profile of MCI varies depending on the underlying etiology and the specific brain regions affected (3). Epidemiological studies indicate that MCI has a global prevalence of 15.56%, with an annual conversion rate to dementia ranging from 8 to 15% (4). Dementia, currently ranked as the seventh leading cause of mortality worldwide, represents a major contributor to disability and dependency among older adults, imposing substantial burdens on both healthcare systems and society at large. Given the progressive nature of cognitive decline and the limited efficacy of current therapeutic interventions for advanced dementia, early identification and preventive strategies for MCI have become crucial priorities in clinical practice and public health policy.

Sarcopenia is a progressive and systemic skeletal muscle disorder characterized by the accelerated loss of muscle mass and function, which significantly impacts physical performance and quality of life (5). Creatinine (Cre), a metabolic byproduct of phosphocreatine in skeletal muscle, is routinely used as a biomarker for kidney function. However, since Cre production is directly proportional to skeletal muscle mass, it also serves as a reliable, cost-effective, and widely accessible surrogate marker for muscle mass in individuals with normal renal function (6, 7). Emerging evidence suggests a potential link between sarcopenia and accelerated cognitive decline, highlighting the interplay between musculoskeletal health and neurodegenerative processes (5, 8). Building on these findings, our current study aims to explore the relationship between circulating creatinine levels and cognitive decline in a Chinese population. This investigation seeks to identify accessible and economical blood-based biomarkers for the early detection of cognitive impairment, which could facilitate timely interventions and improve clinical outcomes.

## Materials and methods

### Study population

**Figure 1:**
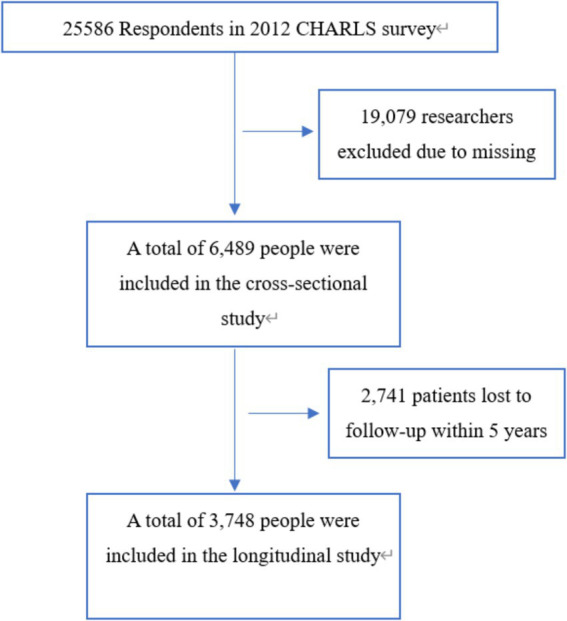
Flow chart of the study sample from the China Health and Retirement Longitudinal Study (CHARLS).

This research was carried out utilizing data from the China Health and Retirement Longitudinal Study (CHARLS) database. CHARLS is a longitudinal survey that tracks a cohort of individuals aged 45 and older, as well as their spouses, in order to provide a representative sample of the Chinese population (9). The first data collection phase of the China Health and Retirement Longitudinal Study (CHARLS) occurred between June 2011 and March 2012, involving biennial computer-assisted personal interviews with participants. The research protocol and implementation were approved by the Biomedical Ethics Review Committee of Peking University (IRB00001052-11015), and all individuals involved in the study gave their informed consent. Additional information can be obtained from the official CHARLS project website. In this study, data from the initial survey and the third follow-up survey (wave 4, 2018) were collected and examined. The study population was rigorously selected through a two-stage screening process using the following exclusion criteria: (1) participants with missing baseline data on cognitive function assessments, serum creatinine levels, or body weight measurements; (2) individuals exhibiting pre-existing cognitive impairment at baseline, as determined by standardized diagnostic criteria. Finally, the cross-sectional analysis included a total of 6,489 participants who met the inclusion criteria, for the longitudinal assessment, we established a final cohort of 3,748 eligible participants after excluding individuals with missing cognitive function data in the fourth wave of follow-up. Figure 1 presents a detailed flowchart illustrating the participant selection process.

### Measurement of cognitive function

Cognitive function was evaluated in a manner consistent with the methodology employed by the American Health and Retirement Study (HRS). A comprehensive assessment was conducted in person, encompassing four key cognitive domains: orientation, memory, computation, and drawing. Orientation and computation were specifically measured using the Telephone Interview for Cognitive Status (TICS). The assessment of orientation involved questions pertaining to temporal awareness, such as the year, month, day, day of the week, and current season, with participants receiving one point for each correct response. In the computation component, individuals were required to perform a series of calculations by subtracting 7 from 100 five times, with one point allocated for each accurate deduction. In the study, each participant was provided with a set of ten words for immediate recollection, and their memory performance was assessed by summing up the scores for immediate and delayed word recall, with one point awarded for each correctly remembered word. Drawing proficiency was evaluated through the replication of a complex image featuring two overlapping pentacle stars, with one point allocated for precise reproduction. The comprehensive cognitive assessment score was derived by aggregating scores from orientation (5 points), computation (5 points), memory (20 points), and drawing (1 point), culminating in a total score of 31 points (10). Cognitive impairment was operationally defined in this study based on the Mini-Mental State Examination (MMSE) scores. Specifically, individuals with no formal education were categorized as cognitively impaired if their MMSE score was less than 18, while those with primary education were considered impaired if their MMSE score was less than 21. Subjects with secondary school education or higher were classified as cognitively impaired if their MMSE score was less than 25. Conversely, individuals with MMSE scores above these thresholds were deemed to have normal cognitive abilities (11).

### Measurement of the Cre/BW ratio

A comprehensive questionnaire was employed to collect data pertaining to demographic characteristics, lifestyle behaviors, and medical history. Height measurements were taken with an accuracy of 0.1 cm, while weight measurements were recorded with a precision of 0.1 kg. Participants were directed to wear light clothing and refrain from wearing shoes during the measurements. The Jaffe kinetic rate technique was utilized to assess Serum Creatinine (Cre) levels. The Cre/BW ratio was calculated by dividing the Cre value in milligrams per deciliter (mg/dL) by the participant’s weight in kilograms.

### Other variables

Other indicators included were age, gender, height, weight, education, smoking and alcohol status, and chronic diseases (hypertension, diabetes, cancer, stroke, heart disease, lung disease, liver disease, and kidney disease).

### Statistical analysis

The study presented continuous variables using means and standard deviations (SD), and categorical variables were represented using numbers and percentages. Between-group comparisons for continuous variables were conducted using the independent sample t-test, while differences among four groups were assessed using one-way analysis of variance (ANOVA). The chi-squared test was employed to identify differences among groups for categorical variables. Unaltered and modified linear regression analyses were utilized to investigate the association between Cre/BW and cognitive function. Subsequently, unaltered and adjusted logistic regression analyses were conducted to assess the connection, with Cre/BW quartiles serving as the independent variables and cognitive function (Q4 vs. Q1) as the dependent variables. Logistic regression analysis was employed to compute the odds ratio (OR) and 95% confidence interval (CI). We utilized univariate and multivariate Cox proportional hazard models to evaluate the association between the Cre/BW ratio and the likelihood of cognitive impairment. The Kaplan–Meier method was employed to conduct survival analysis across the various groups.

To adequately address missing data, we generated 10 imputed data sets with the multiple imputation method by chained equations and reanalyzed such associations. All statistical analyses were performed using IBM SPSS Statistics 26. Statistical differences were considered significant when the calculated *p*-value was less than 0.05.

## Results

### Baseline characteristics of the patients

**Figure 2:**
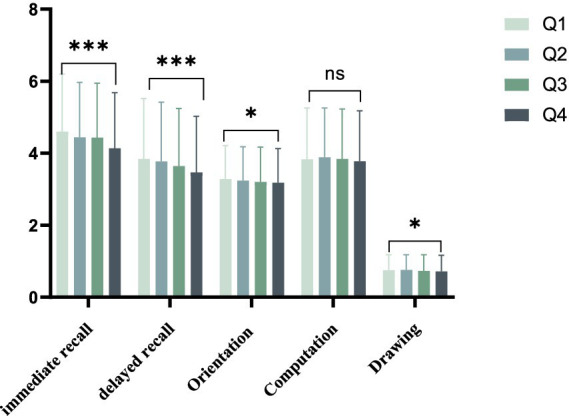
Average cognition scores between groups divided by Cre/BW quartiles. * means *p* < 0.05, *** means *p* < 0.001.

The baseline characteristics of individuals chosen based on quartiles of the Cre/BW ratio are presented in Table 1. A total of 6,489 participants were included in this study. Substantial variations were observed among the four groups with respect to age, body weight, cognitive function scores and past medical history. The analysis of data from the four groups revealed that the Q4 group exhibited a higher average age and lower average weight in comparison to the other groups, with statistical significance observed for all comparisons (*p* < 0.001). Additionally, immediate recall, delayed recall, orientation, computation, and drawing variables examined were lower in the Q4 group relative to the other groups, and this group displayed the lowest average total cognitive function score among the Q4 group (Figure 2).

**Table 1:** Baseline characteristics of study participants according to quartiles of Cre/BW ratio.

	Q1(≤1.086)	Q2 (>1.086 to≤1.290)	Q3 (>1.290 to≤1.538)	Q4 (≥1.538)	*p*-value
Number	1,614	1,614	1,614	1,477	
Age (years)	55.78 ± 7.68	57.32 ± 8.25	59.15 ± 8.51	62.87 ± 9.29	<0.001
Height (cm)	159.70 ± 8.08	159.69 ± 8.39	159.34 ± 8.40	157.88 ± 8.02	<0.001
Weight (kg)	68.50 ± 12.12	62.16 ± 9.69	58.09 ± 8.79	52.85 ± 8.67	<0.001
BMI (kg/m^2^)	26.83 ± 4.27	24.35 ± 3.00	22.83 ± 2.72	21.16 ± 2.75	<0.001
Immediate recall	4.61 ± 1.60	4.44 ± 1.53	4.43 ± 1.52	4.14 ± 1.54	<0.001
Delayed recall	3.84 ± 1.68	3.77 ± 1.65	3.64 ± 1.60	3.47 ± 1.56	<0.001
Orientation	3.28 ± 0.93	3.24 ± 0.94	3.20 ± 0.97	3.18 ± 0.95	0.021
Computation	3.83 ± 1.43	3.89 ± 1.37	3.84 ± 1.39	3.78 ± 1.40	0.149
Drawing	0.75 ± 0.43	0.76 ± 0.42	0.73 ± 0.45	0.72 ± 0.45	0.013
Creatinine	0.63 ± 0.12	0.74 ± 0.11	0.81 ± 0.12	0.97 ± 0.21	<0.001
Gender					<0.001
Man	500 (30.9)	756 (46.6)	908 (56.0)	1,105 (68.2)	
Woman	1,118 (69.1)	865 (53.4)	712 (44.0)	515 (31.8)	
Smoking	473 (27.0)	612 (37.8)	739 (45.6)	910 (56.1)	<0.001
Alcohol drinking status	457 (28.2)	530 (32.7)	663 (41.1)	617 (38.1)	<0.001
Education					
Not receiving education	559 (34.5)	540 (33.3)	599 (37.0)	660 (40.7)	
Primary education	387 (23.9)	388 (23.9)	415 (25.6)	460 (28.4)	
Secondary education and above	675 (41.6)	693 (42.8)	607 (37.4)	501 (30.9)	
Hypertension	490 (30.3)	390 (24.3)	353 (21.9)	342 (21.2)	<0.001
Diabetes	133 (8.3)	105 (6.5)	76 (4.7)	60 (3.7)	<0.001
Cancer	24 (1.5)	17 (1.1)	11 (0.7)	13 (0.8)	0.106
Lung diseases	97 (6.0)	152 (9.4)	167 (10.3)	217 (13.4)	<0.001
Liver diseases	55 (3.4)	62 (3.9)	79 (4.9)	68 (4.2)	0.115
Heart diseases	208 (12.9)	203 (12.6)	165 (10.2)	187 (11.6)	0.073
Stroke	21 (1.3)	30 (1.9)	38 (2.4)	31 (1.9)	0.119
Kidney diseases	91 (5.6)	86 (5.3)	102 (6.3)	147 (9.1)	<0.001

### Linear regression analysis of Cre/BW ratio and cognitive impairment

In order to explore the association between Cre/BW and cognitive function, a linear regression model was constructed. In the unadjusted model, a lower ratio of Cre/BW was found to be positively correlated with better cognitive performance across five domains: orientation, immediate recall, computation, drawing, and delayed recall (*β* = −0.111, 95 CI%: −0.013 to −0.008, *p* < 0.001). Upon controlling for confounding variables, a significant negative correlation was observed between Cre/BW and immediate recall, computation, delayed recall, as well as total cognitive function score (*β* = −0.052, 95 CI%: −0.007 to −0.003, *p* < 0.001) in Table 2.

**Table 2:** Linear regression models for correlations between cre/BW and cognitive function scores.

Outcomes	Model 1		Model 2	
	*β* (95%cl)	*p*	*β* (95%cl)	*p*
Immediate recall	−0.29 (−0.035 ~ −0.023)	<0.001	−0.046 (−0.017 ~ −0.006)	<0.001
Delayed recall	−0.023 (−0.029 ~ −0.018)	<0.001	−0.027 (−0.012 ~ −0.001)	0.026
Orientation	−0.043 (−0.028 ~ −0.007)	<0.001	−0.001 (−0.010 ~ 0.009)	0.919
Computation	−0.032 (−0.016 ~ −0.002)	0.009	−0.042 (−0.018 ~ −0.005)	<0.001
Drawing	−0.037 (−0.054 ~ −0.011)	0.003	0.011 (−0.031 ~ 0.012)	0.365
Total cognitive function score	−0.111 (−0.013 ~ −0.008)	<0.001	−0.052 (−0.007 ~ −0.003)	<0.001

Model 1: Unadjusted model.

Model 2: Adjusted model for age, gender, education, smoking, alcohol consumption, and chronic diseases (hypertension, diabetes, cancer, history of stroke, heart problems, lung disease, liver disease, and kidney disease).

### Logistic regression analysis of Cre/BW ratio and cognitive impairment

In the analysis utilizing total cognitive function scores as a categorical variable (Q4 vs. Q1), a negative association was observed between Cre/BW and cognitive function scores in unadjusted logistic regression models (OR = 0.939, 95% CI: 0.923 to 0.955, *p* < 0.001). This correlation retained its significance even after controlling for potential confounding variables, such as age, gender, education, smoking, alcohol consumption, and chronic diseases (OR = 0.973, 95% CI: 0.951 to 0.996, *p* = 0.019) (Table 3).

**Table 3:** Binary logistic regression models for correlations between Cre/BW and cognitive function.

Outcomes	Model 1		Model 2	
	OR (95%cl)	*p*	OR (95%cl)	*p*
Total cognitive function score (Q2 vs. Q1)	0.989 (0.972–1.006)	0.217	0.988 (0.968–1.008)	0.233
Total cognitive function score (Q3 vs. Q1)	0.965 (0.948–0.983)	<0.001	0.980 (0.959–1.002)	0.068
Total cognitive function score (Q4 vs. Q1)	0.939 (0.923–0.955)	<0.001	0.966 (0.942–0.990)	0.006

Model 1: Unadjusted model.

Model 2: Adjusted model for age, gender, education, smoking, alcohol consumption, and chronic diseases (hypertension, diabetes, cancer, history of stroke, heart problems, lung disease, liver disease, and kidney disease).

### COX survival analysis of Cre/BW ratio and cognitive impairment

Cox proportional hazard models were employed to evaluate the independent effects of the Cre/BW ratio on the risk of developing cognitive impairment, utilizing both univariate and multivariate analyses. The results, as detailed in Table 4, include effect sizes represented by HR and 95% CI. The unadjusted model indicated a positive correlation between the Cre/BW ratio and the incidence of cognitive impairment, yielding an (HR = 1.222, 95% CI: 1.117 to 1.336, *p* < 0.001). Following adjustments for variables such as age, sex, height, BMI, alcohol consumption, smoking status, and chronic diseases, the multivariate analysis maintained this positive association, with an (HR = 1.207, 95% CI: 1.073 to 1.359, *p* = 0.002). Furthermore, participants in the highest quartile of the Cre/BW ratio exhibited an increased likelihood of developing cognitive impairment compared to those in the lowest quartile (HR = 1.118, 95% CI: 1.048 to 1.346, *p* = 0.007).

**Table 4:** Relationship between Cre/BW ratio and incident cognitive impairment in different models.

Variable	Model 1		Model 2	
	HR (95% CI)	*p* value	HR (95% CI)	*p* value
Cre/BW ratio (×100)	1.222 (1.117, 1.336)	<0.001	1.207 (1.073, 1.359)	0.002
Q1	Ref	Ref	Ref	Ref
Q2	1.011 (0.923, 1.107)	0.819	0.993 (0.901, 1.095)	0.886
Q3	1.065 (0.971, 1.168)	0.184	1.047 (0.940, 1.166)	0.402
Q4	1.216 (1.106, 1.337)	<0.001	1.188 (1.048, 1.346)	0.007

Model 1: Unadjusted model.

Model 2: Adjusted model for age, gender, height, BMI, smoking, alcohol consumption, and chronic diseases (hypertension, diabetes, cancer, history of stroke, heart problems, lung disease, liver disease, and kidney disease).

**Figure 3:**
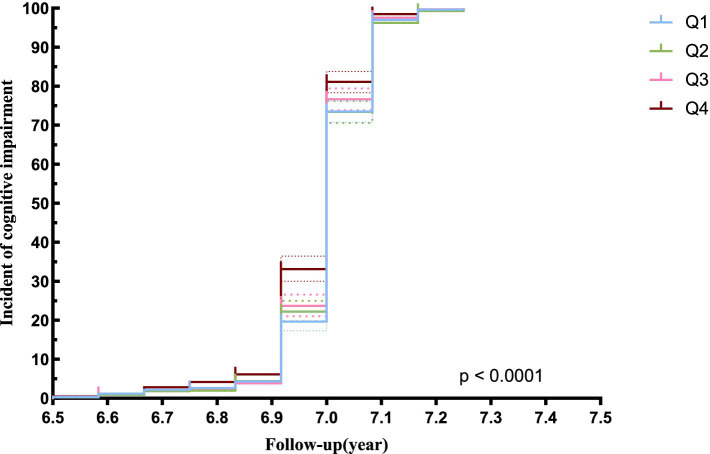
Kaplan–Meier analysis of cognitive impairment risk according to Cre/Bw ratio.

The Kaplan–Meier curve analysis indicated a significant difference in the cumulative risk of developing cognitive impairment across the quartiles of the Cre/BW ratio (logrank test, *p* < 0.0001). Furthermore, the risk exhibited a gradual increase corresponding to higher Cre/BW ratios, culminating in the highest risk of cognitive impairment observed in the upper quartile (Figure 3).

## Discussion

Sarcopenia is a geriatric syndrome characterized by the progressive and generalized loss of skeletal muscle mass and function, which is strongly associated with increased morbidity, mortality, and reduced quality of life (12). Based on the most widely accepted diagnostic criteria, the prevalence of sarcopenia is estimated to range from 5 to 13% in individuals aged 60–70 years, escalating significantly to 11–50% in those over 80 years of age. Alarmingly, epidemiological projections suggest that the global burden of sarcopenia could affect up to two billion individuals by 2050, underscoring its growing public health significance (12). The diagnosis of sarcopenia requires a comprehensive evaluation of three key components: muscle mass, muscle strength, and physical performance, as recommended by international consensus guidelines (13).

Cre is a well-established biomarker for skeletal muscle mass, demonstrating a strong positive correlation with the total quantity of skeletal muscle in the body. Studies have shown that the body mass-adjusted skeletal muscle mass index (SMI) is positively associated with the creatinine-to-body weight ratio (Cre/BW). Notably, prior research utilizing Cre/BW as a surrogate measure of muscle mass has identified significant associations between sarcopenia and an increased risk of non-alcoholic fatty liver disease (NAFLD) and diabetes mellitus. Building on these findings, we sought to investigate the relationship between sarcopenia and cognitive impairment, employing Cre/BW as a practical and accessible proxy for muscle mass. In this study, we observed that lower Cre/BW levels were independently associated with better cognitive performance, as evidenced by both linear and logistic regression analyses after adjusting for potential confounders. These results align with previous studies demonstrating a link between sarcopenia and accelerated cognitive decline, further underscoring the interplay between musculoskeletal health and neurodegenerative processes (8, 14–16). Our study revealed a significant inverse association between Cre/BW and cognitive function. Cross-sectional analysis demonstrated that Cre/BW was negatively correlated with cognitive performance scores, and this relationship remained robust after comprehensive adjustment for potential confounders, including age, gender, educational attainment, smoking status, alcohol consumption, and comorbidities. To elucidate the potential causal relationship between Cre/BW and cognitive decline, we performed longitudinal analyses, which identified Cre/BW as an independent risk factor for mild cognitive impairment (MCI). Notably, in the fully adjusted model, participants in the highest quartile of Cre/BW showed a 1.18-fold increased risk of developing MCI compared to those in the lowest quartile. These findings are consistent with existing literature documenting the association between sarcopenia and cognitive impairment. Considering the multifactorial nature of sarcopenia diagnosis, which requires comprehensive multidimensional assessment, our study provides novel evidence that Cre/BW, as a surrogate marker for sarcopenia, demonstrates significant concordance with previous investigations in this field.

The estimated prevalence of MCI in individuals aged 65 years and older ranges from 3 to 22% across different populations and diagnostic criteria (1, 17). Accumulating evidence from recent epidemiological studies underscores the critical importance of early detection and accurate diagnosis of MCI. Early identification of cognitive decline enables timely implementation of targeted interventions, which may not only delay the progression to dementia but also potentially prevent further neurodegeneration. This therapeutic window, occurring before substantial neurological damage has accumulated, represents a crucial opportunity for implementing effective preventive strategies and preserving cognitive function in aging populations (18, 19). Current diagnostic approaches for MCI primarily rely on neuropsychological assessments, particularly the Brief Cognitive Assessment Tool, due to the lack of validated biomarkers or reliable hematological indicators for routine clinical use (20, 21). Our study provides novel insights through the identification of both cross-sectional and longitudinal associations between the Cre/BW and MCI, suggesting its potential utility as a quantitative marker for early detection of cognitive impairment. Furthermore, we established a significant correlation between sarcopenia and MCI, supported by robust epidemiological evidence. These findings not only advance our understanding of the pathophysiological link between musculoskeletal health and cognitive function but also pave the way for developing targeted prevention strategies in at-risk older populations.

The precise pathophysiological mechanisms underlying sarcopenia and MCI remain incompletely understood. Emerging evidence suggests (22–25) that estrogen deficiency may represent a shared etiological factor contributing to both osteoporosis and cognitive decline. As a pleiotropic hormone, estrogen plays crucial roles not only in maintaining bone homeostasis but also in neuroprotection. The decline in estrogen levels, particularly during the postmenopausal period, has been associated with accelerated bone loss and cognitive dysfunction. Mechanistic studies have demonstrated that estrogen exerts neuroprotective effects through multiple pathways, including the suppression of neuronal apoptosis, attenuation of oxidative stress, and modulation of neuroinflammatory responses (26).

Emerging evidence suggests that inflammatory pathways and metabolic dysregulation may serve as critical mediators in the bidirectional relationship between osteoporosis and cognitive impairment (27). Chronic low-grade inflammation, characterized by persistent elevation of pro-inflammatory cytokines (e.g., IL-6, TNF-*α*), and metabolic syndrome components, including insulin resistance, hypertension, and dyslipidemia, have been identified as shared risk factors for both conditions. A meta-analysis reveal a significant co-occurrence of systemic inflammation and metabolic disturbances in osteoporotic patients, with these pathological processes potentially synergistically accelerating cognitive decline through multiple mechanisms (23).

Nutritional status and lifestyle factors have emerged as potentially modifiable elements in the complex interplay between osteoporosis and cognitive impairment (28). Epidemiological studies demonstrate that suboptimal nutrition represents a significant shared risk factor for both conditions, particularly in aging populations (29, 30). Current evidence suggests that adequate caloric intake and optimal protein consumption (1.0–1.2 g/kg body weight/day) are crucial for maintaining musculoskeletal health and cognitive function in older adults (31). Furthermore, micronutrient deficiencies, particularly in vitamin D, calcium, and B vitamins, have been implicated in the pathogenesis of both bone loss and cognitive decline (32–34).

Additionally, some research suggests that exercise-induced myokines may play a pivotal role in mediating the beneficial effects of physical activity on cognitive function. Skeletal muscle, when activated through regular physical activity, functions as an endocrine organ, secreting various cytokines and neurotrophic factors, collectively termed myokines. These myokines can cross the blood–brain barrier and exert neuroprotective effects through multiple mechanisms, including the promotion of neurogenesis, enhancement of synaptic plasticity, and reduction of neuroinflammation (35, 36).

This research possesses several notable strengths. Firstly, the employment of a nationally representative longitudinal survey of older adults in China enhances the robustness of the extrapolation of findings. Secondly, this study is pioneering in its investigation of the association between cognitive impairment and the variables of Cre/BW, utilizing both cross-sectional and longitudinal analyses while controlling for various confounding factors.

Despite its contributions, our study has several limitations that warrant careful consideration. First, the generalizability of our findings may be limited, as the study population exclusively comprised Chinese participants. This restricts the extrapolation of our results to other ethnic groups and underscores the need for validation in more diverse populations. Second, our analysis did not account for certain potential confounding factors, such as nutritional status and physical activity levels. Third, the absence of baseline data necessitated the exclusion of a substantial number of participants, potentially introducing selection bias, despite our efforts to mitigate this through sensitivity analyses. Fourth, while the longitudinal analysis demonstrated a more robust association between the Cre/BW and MCI compared to the cross-sectional analysis, the precise biological mechanisms underlying this relationship remain elusive. To address these limitations, further experimental studies are warranted to validate the observed association and elucidate the causal pathways linking skeletal muscle health to cognitive decline.

## Conclusion

In summary, we utilized the CHARLS database to investigate the relationship between Cre/BW levels and cognitive function among the middle-aged and older population in China, thereby contributing new evidence to support a causal link between these variables.

## Data Availability

Publicly available datasets were analyzed in this study. This data can be found at: China Health and Retirement Longitudinal Study (https://charls.pku.edu.cn/).
